# Cloud Computing for Protein-Ligand Binding Site Comparison

**DOI:** 10.1155/2013/170356

**Published:** 2013-05-16

**Authors:** Che-Lun Hung, Guan-Jie Hua

**Affiliations:** ^1^Department of Computer Science and Communication Engineering, Providence University, Taiwan Boulevard, Shalu District, Taichung 43301, Taiwan; ^2^Department of Computer Science and Information Engineering, Providence University, Taiwan Boulevard, Shalu District, Taichung 43301, Taiwan

## Abstract

The proteome-wide analysis of protein-ligand binding sites and their interactions with ligands is important in structure-based drug design and in understanding ligand cross reactivity and toxicity. The well-known and commonly used software, SMAP, has been designed for 3D ligand binding site comparison and similarity searching of a structural proteome. SMAP can also predict drug side effects and reassign existing drugs to new indications. However, the computing scale of SMAP is limited. We have developed a high availability, high performance system that expands the comparison scale of SMAP. This cloud computing service, called Cloud-PLBS, combines the SMAP and Hadoop frameworks and is deployed on a virtual cloud computing platform. To handle the vast amount of experimental data on protein-ligand binding site pairs, Cloud-PLBS exploits the MapReduce paradigm as a management and parallelizing tool. Cloud-PLBS provides a web portal and scalability through which biologists can address a wide range of computer-intensive questions in biology and drug discovery.

## 1. Introduction

By virtue of its 3D structure, a protein performs thousands of life-critical functions at the molecular level. Detection and characterization of protein structural ligand binding sites and their interactions with binding partners are pivotal to a wide range of structure-function correlation problems—predicting functions for structural genomics targets, identifying and validating drug targets, prioritizing and optimizing drug leads, and correlating molecular functions to physiological processes in drug design [1].

Xie et al. [2–4] proposed an efficient and robust algorithm called SMAP, which quantitatively characterizes the geometric properties of proteins. Ligand binding sites predicted by SMAP have been experimentally validated [4–7]. SMAP has also been applied to drug design problems, such as constructing drug-target interaction networks [4], designing polypharmacology drugs [5], assigning old drugs to new indications [6], and predicting the side effects of drugs [8, 9]. The web service tool SMAP-WS [1] implements SMAP via Opal [10]. Although the parallel implementation of SMAP improves the speed of database searching, it cannot operate at the scale and availability demanded by current Internet technology.

Recently, an Internet service concept known as cloud computing has become popular for providing various services to users. The cloud computing environment is a distributed system with extremely scalable IT-related capabilities, providing multiple external customers with numerous services. Cloud computing also enables the copying of vast datasets to many users with high fault tolerance. Another popular open-source software framework designed for data-intensive distribution is Hadoop [11]. This framework processes petabytes of data intercepting thousands of nodes. Hadoop provides the MapReduce programming model, by which parallel computing of large data sets can be implemented in the cloud computing environment. MapReduce enables distributed computing of the mappers and reducers. Each mapper performs an independent map operation which is parallelized with the tasks of other mappers. Similarly, a set of reducers can perform a set of reduce operations. All outputs of the map operations possessing the same key are presented to the same reducer at the same time. Two additional important benefits of Hadoop are scalability and fault tolerance. Hadoop can guide jobs toward successful completion even when individual nodes or network components experience high failure rates. Meanwhile, a machine can be readily attached as a mapper and reducer in the Hadoop cluster. The Hadoop platform, therefore, is regarded as a superior solution to real-world data distribution problems. To date, Hadoop has been applied in a range of bioinformatics domains [12–16]. 

Cloud computing platforms are usually based on virtualization technology. Computing resources are combined or divided into one or more operating environments using methodologies such as hardware and software partitioning or aggregation, partial or complete machine simulation, and emulation and time sharing. A virtual machine (VM) is a machine simulation created by virtualization technology, which resides in a physical machine and shares its physical resources. The web service Amazon Elastic Compute Cloud (Amazon EC2) [17] uses virtualization technology to generate resizable computing capacity in the cloud. The service provides a true virtual computing environment, allowing users to launch VMs with a variety of operating systems. Users can construct their own elastic cluster systems by attaching or removing VMs.

In this paper, we combine three technologies, Hadoop framework, virtualization, and SMAP, to develop a cloud computing service for structural ligand binding site comparison. Each mapper or reducer in the cloud platform is a VM. The platform uses MapReduce to simultaneously process numerous comparison jobs. Similarly, the number of VMs can be adjusted to the size of the comparison job (large and small jobs demand more and fewer VMs, resp.). Hadoop enables our cloud platform to recover the comparison job from a crashed VM or physical machine by reassigning the job to a healthy VM or a physical machine. The cloud platform can achieve high performance, scalability, and availability. The experimental results demonstrate that applying the Hadoop framework on a virtualization platform enhances the computational efficiency of the proposed service. The cloud service is available at http://bioinfo.cs.pu.edu.tw/cloud-PLBS/index.html.

## 2. Method

Cloud-PLBS is a robust, elastic cloud computing service for protein-ligand binding site comparison. It guarantees rapid return of comparison results. Cloud-PLBS embraces three technologies, virtualization, Hadoop, and SMAP, used to build the cloud computing infrastructure, perform parallel computation, and compare ligand binding sites, respectively.

### 2.1. Structural Proteome-Wide Ligand Binding Site Comparison

SMAP is an efficient and robust algorithm that performs pair-wise comparison of two potential ligand binding sites. The user enters two protein structure IDs, and SMAP downloads the relevant protein structures from the RCSB Protein Data Bank (PDB) [18]. Protein structure binding sites are compared in four stages.

*Step 1*. The protein structures are represented by C-*α* atoms for structural variation tolerance.

*Step 2*. Amino acid residues are characterized by surface orientation and a geometric potential.

*Step 3*. Protein structures are compared using a sequence order-independent profile-profile alignment (SOIPPA) algorithm.

*Step 4*. Similarity between two binding sites is determined through the combination of geometrical fit, residue conservation and physiochemical similarity.

In Cloud-PLBS, each paired protein structure comparison is regarded as an SMAP job. Each SMAP job compares two ligand binding sites by the four stages listed above. 

### 2.2. Cloud-PLBS by Combining Hadoop and Virtualization

**Figure 1:**
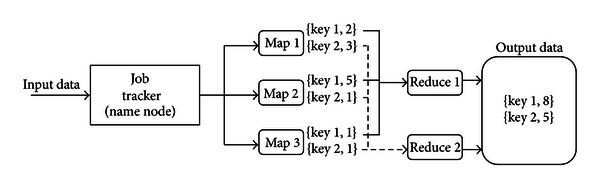
Map/reduce framework of Hadoop.

As mentioned above, Cloud-PLBS comprises Hadoop, virtualization, and SMAP. Hadoop coordinates computing nodes to parallelize distributed data. Parallel computing applications are developed via the map/reduce parallel programming model. The standard map/reduce mechanism has been applied in many successful cloud computing service providers, such as Yahoo, Amazon EC2, IBM, and Google. The map/reduce framework of Hadoop is illustrated in Figure 1. Input data are divided into smaller chunks corresponding to the number of mappers. The mapper stage output is formatted as 〈key, value〉 pairs. Output from all mappers is classified by key before being distributed to the reducer. The reducer then combines the keyed values. Its output is also formatted as 〈key, value〉 pairs, where each key is unique.

**Figure 2:**
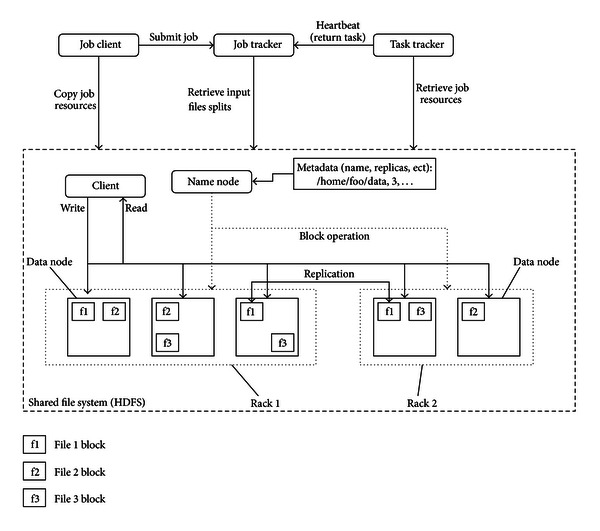
The architecture of Hadoop cluster.

The Hadoop cluster includes a single master and multiple slave nodes. The master node comprises a job tracker, task tracker, name node and data-node. A slave node, or computing node, consists of a data node and task tracker. The job tracker distributes map/reduce tasks to computing nodes within the cluster, ideally those already containing the data, or at least within the same rack. A task tracker node accepts map, reduce and shuffle operations from the job tracker. The architecture of the Hadoop cluster is shown in Figure 2. 

Hadoop Distributed File System (HDFS) is the primary file system used by the Hadoop framework. Each input file is split into data blocks that are distributed to data nodes. Hadoop also creates multiple replicas of data blocks and distributes them to data nodes throughout a cluster, ensuring reliable, extremely rapid computations. The name node serves as both a directory namespace manager and a node metadata manager for the HDFS. The HDFS architecture operates on a single name-node. 

**Figure 3:**
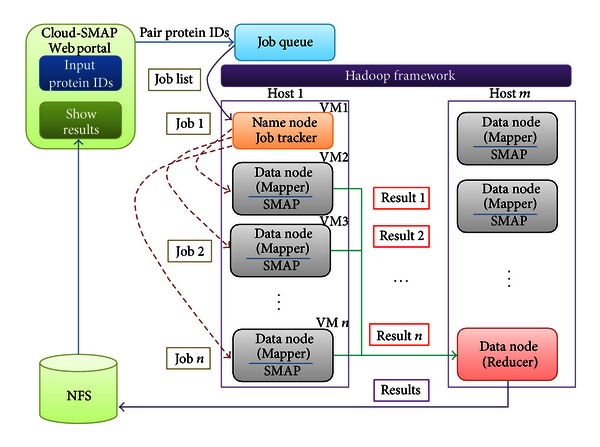
The cloud platform of Cloud-PLBS.

Resource capacity permitting virtualization technology can host several virtual machines within a physical machine. The proposed cloud service platform combines Hadoop and virtualization technology, such that all nodes of the Hadoop cluster reside in VMs. The cloud computing architecture of Cloud-PLBS is illustrated in Figure 3. As shown in that figure, master node (name node) and slave node (data node) constitute the master VM and slave VM, respectively. Submitted SMAP jobs are recorded in a job queue. The master node periodically obtains SMAP jobs from the job queue and assigns them to slave nodes; a slave node (or mapper) performs the task. Once all of the SMAP jobs are complete, the reducer collects the comparison results from all mappers and stores them in the Network File System (NFS) storage. A single comparison result is stored in a single file in NFS. This architecture imbues Cloud-PLBS with three desirable characteristics: high performance, scalability, and availability. 

#### 2.2.1. High Performance

 In Cloud-PLBS, the SMAP jobs are performed in parallel by the map/reduce framework. The number of SMAP jobs that can be performed simultaneously is the number of data nodes. If the number of SMAP jobs exceeds the number of data nodes, the number node assigns the remaining jobs as soon as a data node becomes available.

#### 2.2.2. Availability

In the event of system failure, Cloud-PLBS continues performing SMAP jobs via the Hadoop fault tolerance mechanism. When a data node (mapper) fails during SMAP computation, name node reassigns its job to another slave node (mapper). Therefore, in Cloud-PLBS, all of the submitted SMAP jobs are executed in the event of data node failure. A hardware failure on the physical server will terminate all virtual machines running on it. In this more catastrophic event, SMAP jobs can be reassigned to several new virtual machines created on available hosts. As a result of this operation, Cloud-SMAP has high availability. 

#### 2.2.3. Scalability

 If excessively many SMAP jobs are submitted, Cloud-PLBS can create new slave VMs as data nodes to accept more jobs, leading to enhanced performance. New VMs are easily created in the Cloud-PLBS architecture. At the same time, redundant VMs can be destroyed to preserve physical resources. 

## 3. Cloud-PLBS Platform

Cloud-PLBS is a software (SaaS) as a service service operating under the Hadoop framework and virtualization technology. The cloud computing platform is composed of an NFS server and four IBM blade servers in the Providence University Cloud Computation Laboratory. Each server is equipped with two Quad-Core Intel Xeon 2.26 GHz CPUs, 24 G RAMs, and 296 G disks. Each server can accommodate 8 virtual machines; each virtual machine is set to one core CPU, 2 G RAM, and 30 G disk running under the Ubuntu operating system version 10.4 with Hadoop version 0.2 MapReduce framework. Each virtual machine is responsible for a map operation and a reduce operation. Therefore, up to eight map/reduce operations may be undertaken. 

**Figure 4:**
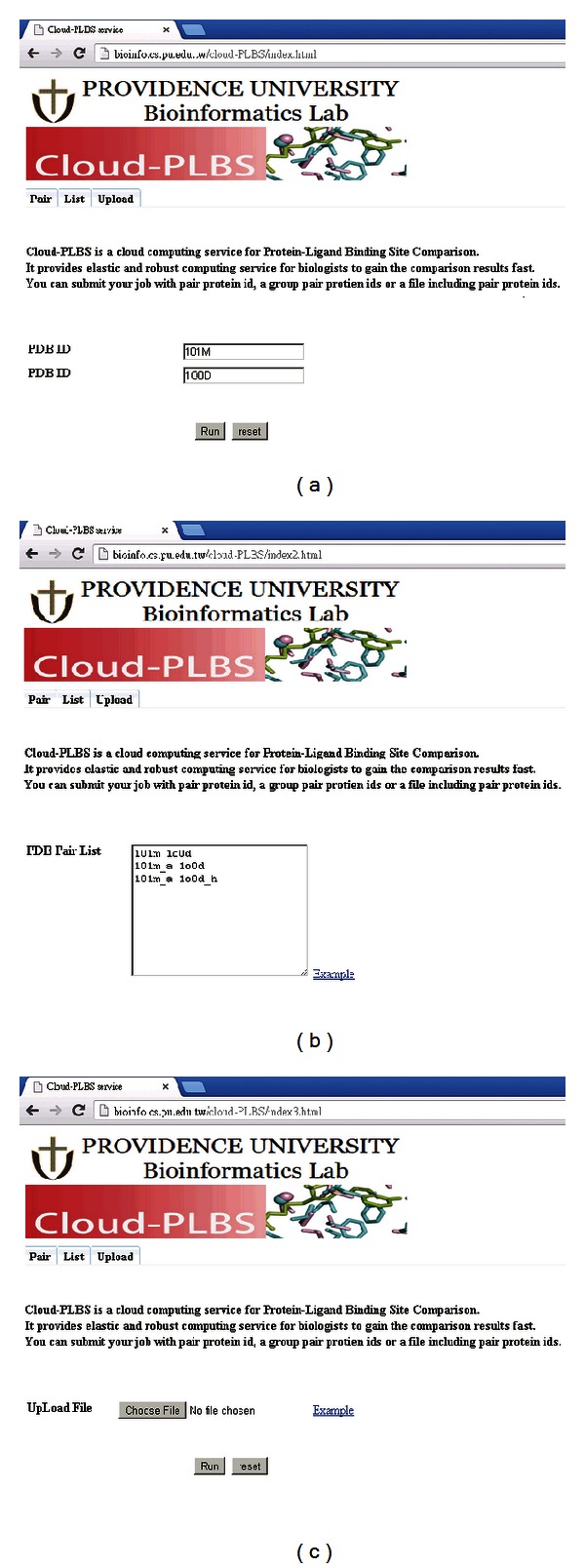
Web portal of Cloud-PLBS for entering protein IDs. (a) Two protein IDs. (b) List of paired protein IDs. (c) Upload file.

**Figure 5:**
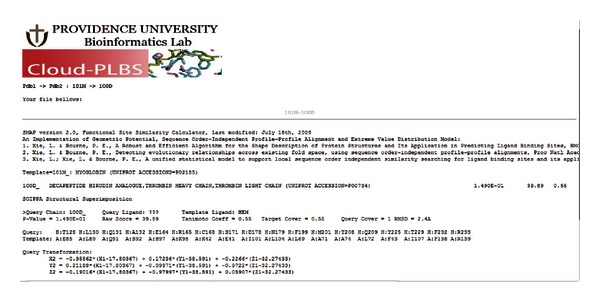
The result produced by Cloud-PLBS. The protein IDs are 101 M and 100D.

Figure 4 shows the web portal of Cloud-PLBS. Data may be entered in three ways: by entering two protein IDs (Figure 4(a)), by listing several pairs of protein IDs (Figure 4(b)), or by uploading containing paired protein IDs (Figure 4(c)). All of these pair protein IDs are recorded in a job queue upon submission. The name node (mater node) extracts the paired protein IDs from the queue, and assigns individual SMAP jobs to data nodes (slave nodes).  Figure 5 shows the results of comparisons produced by Cloud-PLBS. 

## 4. Performance Evaluation

**Figure 6:**
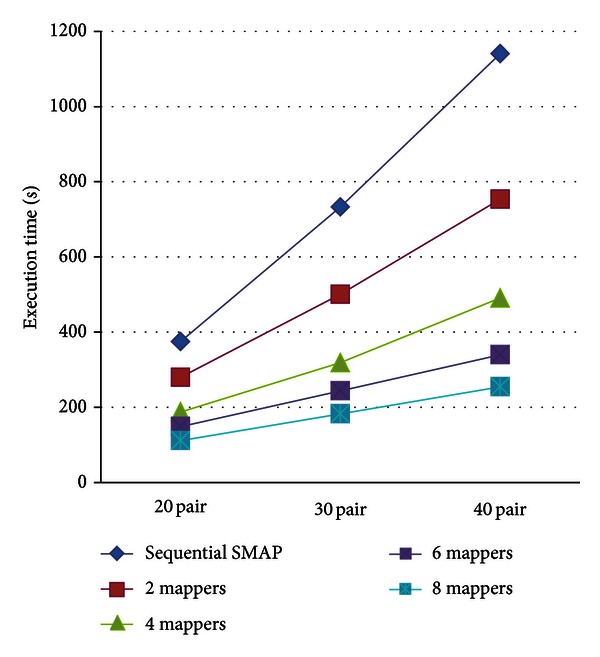
Performance of sequential SMAP program and Cloud-PLBS using 2, 4, 6, and 8 mappers.

**Figure 7:**
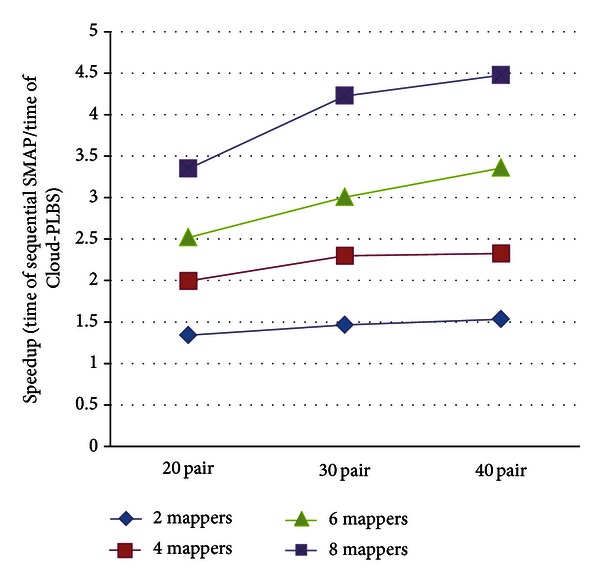
Execution speed of sequential SMAP program and Cloud-PLBS using 2, 4, and 6 mappers.

To assess the performance of the proposed cloud service, we compared the execution time between stand-alone SMAP and Cloud-PLBS. The performance of both programs depends upon the number of SMAP jobs (the number of paired protein IDs) and the number of computing nodes (the number of VMs). Therefore, the performance between the programs is tested with respect to these two factors. The results are shown in Figure 6. As shown in the figure, the execution time of 20 protein pairs (jobs) can be reduced from 375 seconds (consumed by the sequential SMAP program) to 280 seconds, 188 seconds, 149 seconds, and 112 seconds by executing Cloud-PLBS with 2, 4, 6, and 8 mappers, respectively. Given 20, 30 and 40 protein pairs, Cloud-PLBS with 2, 4, and 6 and 8 mappers saves roughly 30%, 54%, 66%, and 74% execution time (relative to sequential SMAP) in average, respectively (see Table 1).  Figure 7 demonstrates the enhanced speed achieved by Cloud-PLBS using different numbers of mappers. Clearly, the execution time is effectively reduced when more than two mappers are involved. In general, more mappers (VMs) achieve a faster processing speed.

**Figure 8:**
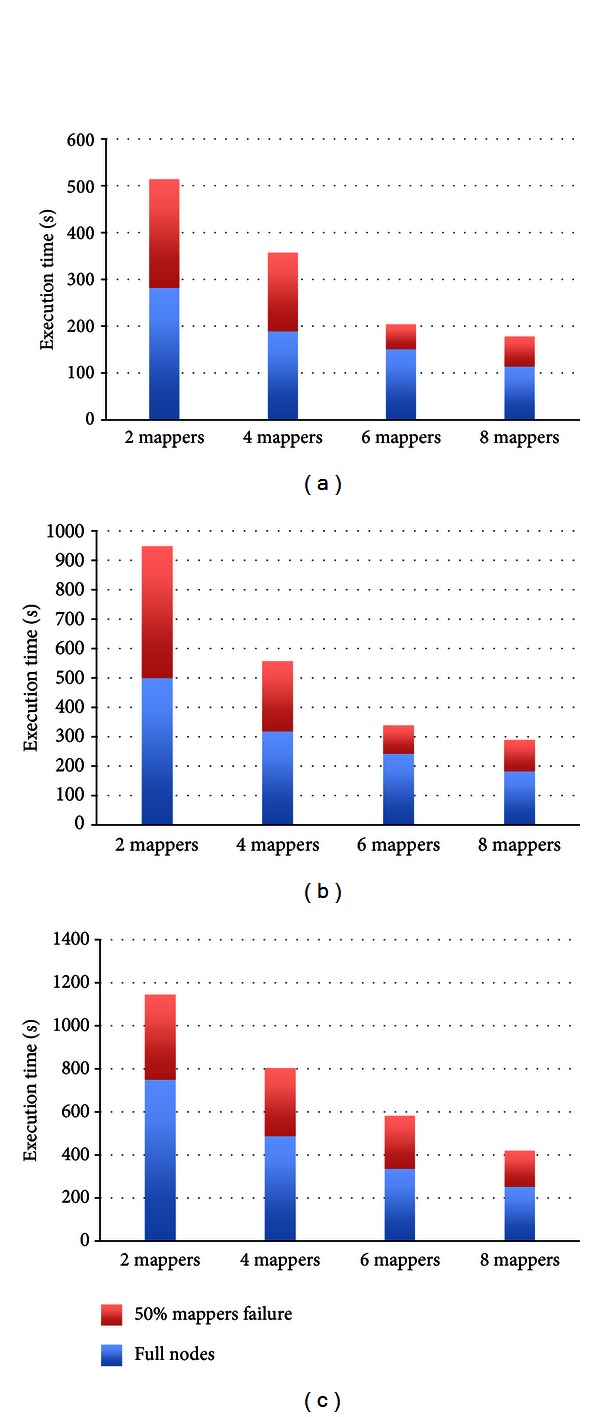
Execution time of a half of node failure of Cloud-PLBS. (a) 20 pair (b) 30 pair (c) 40 pair.

To evaluate the reliability and availability of the proposed cloud service, we performed a simulation to observe the performance when mappers fail. In this simulation, half of the mappers failed in the duration of executing SMAP. According to the features of Hadoop, the computing process at the failed node is able to continue at another node that has the replica of data of the failed node. In this simulation, the heartbeat time is set to one minute, and the number of replica is set to three as default. Therefore, all of jobs can be completed even when some of the nodes fail. Figures 8(a), 8(b), and 8(c) demonstrate the performance between the different number of nodes meeting corresponding half of nodes fail for processing 20 pair, 30 pair, and 40 pair data set, respectively. The execution time with no failure is shown as the blue bar, and the execution time with failure in a half of nodes is the sum of blue bar and red bar which is extra time when failure occurrence. From the experiment results, it shows that the jobs can be completed less than the double successful execution time in the proposed service. Although half of the nodes fail, the execution time of redundancy is related to the number of nodes too. There are extra 165 seconds for 8 mappers, 263 seconds for 6 mappers, 313 seconds for 4 mappers, and 391 seconds for 2 mappers when half of the nodes fail Occurs, respectively. Thereby, our cloud service is node failure-free. 

## 5. Conclusion

The detection and characterization of protein ligand binding sites and their interactions with binding partners are an essential component of modern drug design. The software tool SMAP was designed to achieve these goals. Although SMAP outperforms most existing ligand binding site comparison tools, it cannot achieve the high scalability and availability demanded by huge database searching. 

In this paper, we exploit the new internet service concept known as cloud computing. The proposed cloud computing service is called Cloud-PLBS (where PLBS denotes protein-ligand binding site). The platform integrates the Hadoop framework, virtualization technology, and SMAP tool to guarantee high performance, availability, and scalability. Cloud-PLBS ensures that all submitted jobs are properly completed, even on a large cloud platform where individual nodes or network components are prone to failure. We experimentally verified that the platform is computationally more efficient than standard SMAP. Therefore, it presents as a desirable tool for analyzing protein structure and function under reasonable time constraints.

## Figures and Tables

**Table 1:** Execution time and proportional reduction (relative to sequential SMAP) of Cloud-PLBS using different numbers of mappers.

Method	20 pairs		30 pairs		40 pairs	
	Execution time (sec)	Reduction rate	Execution time (sec)	Reduction rate	Execution time (sec)	Reduction rate
Sequential SMAP	375		733		1141	
Cloud-PLBS for 2 mappers	280	24.44%	501	31.66%	754	33.92%
Cloud-PLBS for 4 mappers	188	48.87%	319	56.49%	491	56.97%
Cloud-PLBS for 6 mappers	149	60.27%	244	66.72%	340	70.21%
Cloud-PLBS for 8 mappers	112	70.13%	183	75.03%	255	77.65%
